# Transmission of a heterologous clade C *Symbiodinium* in a model anemone infection system via asexual reproduction

**DOI:** 10.7717/peerj.2358

**Published:** 2016-08-24

**Authors:** Wan-Nan U. Chen, Ya-Ju Hsiao, Anderson B. Mayfield, Ryan Young, Ling-Lan Hsu, Shao-En Peng

**Affiliations:** 1 Department of Biological Science and Technology, I-Shou University, Kaohsiung, Taiwan; 2 National Museum of Marine Biology and Aquarium, Checheng, Pingtung, Taiwan; 3 Living Oceans Foundation, Landover, MD, United States of America; 4 University of California, Davis, United States; 5 Department of Life Science, National Taiwan University, Taipei, Taiwan; 6 Graduate Institute of Marine Biology, National Dong Hwa University, Checheng, Pingtung, Taiwan

**Keywords:** Endosymbiology, Marine biology, Microalgae

## Abstract

Anemones of genus *Exaiptasia* are used as model organisms for the study of cnidarian-dinoflagellate (genus *Symbiodinium*) endosymbiosis. However, while most reef-building corals harbor *Symbiodinium* of clade C, *Exaiptasia* spp. anemones mainly harbor clade B *Symbiodinium* (ITS2 type B1) populations. In this study, we reveal for the first time that bleached *Exaiptasia pallida* anemones can establish a symbiotic relationship with a clade C *Symbiodinium* (ITS2 type C1). We further found that anemones can transmit the exogenously supplied clade C *Symbiodinium* cells to their offspring by asexual reproduction (pedal laceration). In order to corroborate the establishment of stable symbiosis, we used microscopic techniques and genetic analyses to examine several generations of anemones, and the results of these endeavors confirmed the sustainability of the system. These findings provide a framework for understanding the differences in infection dynamics between homologous and heterologous dinoflagellate types using a model anemone infection system.

## Introduction

The sea anemone *Exaiptasia pallida* is a widespread species that has been well-adopted as a model animal for the study of cnidarian endosymbiology, particularly with associations featuring the dinoflagellate algae *Symbiodinium* sp. (Weis et al., 2008; Grajales & Rodriguez, 2016). In the laboratory, bleached anemones can be prepared by cold shock treatment (Muscatine, Grossman & Doino, 1991) and then maintained for several years in laboratory culture. Recently, genetic examinations of field-collected specimens and laboratory infection demonstrate that *E. pallida* anemones primarily harbor *Symbiodinium* spp. of *Symbiodinium minutum* (ITS2 type B1) and *Symbiodinium* A4 (ITS2 type A4), and in rare cases, a mixed population of *Symbiodinium* B1 and C1 (Thornhill et al., 2013; Grajales, Rodriguez & Thornhill, 2016), which can be readily isolated from these anemones and cultured *in vitro* (Kinzie III et al., 2001; Wang et al., 2008; Peng et al., 2012; Xiang et al., 2013). By infecting bleached anemones with free-living *Symbiodinium*, the endosymbiotic relationship can then be re-established and tracked in order to understand the recognition processes that occur at the molecular level and culminate in successful mutualisms (Weis et al., 2008; Kinzie & Chee, 1979; Lin, Wang & Fang, 2000; Chen et al., 2004; Hong et al., 2009; Wang et al., 2013; Hambleton et al., 2014; Xiang et al., 2013).

In our previous research involving the infection of *E. pallida* anemones with various *Symbiodinium* sp., we discovered the uptake and consequent cellular proliferation of a cultured *Symbiodinium* of clade C, a lineage of dinoflagellates known to predominantly infect reef corals (Chen et al., 2005; LaJeunesse et al., 2003; LaJeunesse et al., 2004a; LaJeunesse et al., 2008; LaJeunesse et al., 2010; Lien, Fukami & Yamashita, 2012). This is a particularly interesting finding, and it needs to be confirmed if this *Exaiptasia*-clade C *Symbiodinium* association is a sustainable endosymbiotic relationship. Furthermore, if the nature of this association is proven to be similar to that of corals, this *Exaiptasia*-clade C *Symbiodinium* association deserves even more merit as a model system for understanding reef corals, which cannot be successfully bleached and re-infected due to the stress it imposes on the corals (i.e., they are obligately endosymbiotic).

Metabolic relationships between corals and *Symbiodinium* are functionally diverse, depending largely on the genetic identity of the latter (Baker et al., 2004; Abrego et al., 2008; Stat, Morris & Gates, 2008; Yuyama, Harii & Hidaka, 2012; Yuyama & Higuchi, 2014). For instance, corals associated with *Symbiodinium* of clade D have been shown to possess an enhanced degree of thermal tolerance (Baker et al., 2004). Understanding the physiological consequences of engaging in an endosymbiotic relationship with dinoflagellates of differing identity would then be useful in formulating predictions as to how anemones, or even reef corals, may respond to global climate change. To further corroborate our previously unpublished findings and gain greater insight into the ability to develop a heterologous anemone-*Symbiodinium* infection system, we co-cultured exogenously supplied *Symbiodinium* C1 with bleached anemones (infection trial). The *Symbiodinium* C1-infected anemones were then maintained in the laboratory for more than one year, and asexual reproduction (pedal laceration) of *Symbiodinium* C1-infected anemones was observed.

## Materials and Methods

### Preparation of clade C *Symbiodinium*-infected anemones

The sea anemones, *Exaiptasia pallida*, were collected from the tanks in the Husbandry Centre of the National Museum of Marine Biology and Aquarium. The origin of the anemones comes from the location beneath the native habitat (N22 03 00.08 E120 41 42.88) of *Exaiptasia pallida*, which harbor *Symbiodinium minutum* (ITS2 type B1) (Grajales & Rodriguez, 2014; Grajales & Rodriguez, 2016; Grajales, Rodriguez & Thornhill, 2016). Clade C *Symbiodinium* (CCMP 2466) were purchased from the National Center for Marine Algae and Microbiota (NCMA), which were originally isolated from the corallimorph *Discosoma sanctithomae* in the Caribbean Sea (https://ncma.bigelow.org/ccmp2466). The *Symbiodinium* were cultured in the laboratory according to a previously published protocol (Peng et al., 2012) for several years, and its genetic identity was confirmed to be ITS2 type C1 (Fig. S1; Krueger et al., 2015). Briefly, *Symbiodinium* cells were cultured in Guillard’s (f/2) media (without silica, Cat. G0154, Sigma-Aldrich, USA) containing antibiotics (10 mg ml^−1^ streptomycin and 10 units ml^−1^ penicillin; Cat. 15140-122, Gibco, USA) and maintained at 25 °C with a photoperiod of 12 h light (∼40 µmol m^−2^ s^−1^): 12 h dark (12L/12D). The *Symbiodinium* cultures were changed every week. The infection trials were performed by collecting f/2 media-cultured clade C *Symbiodinium* in the early stationary phase via centrifugation (800 *xg* for 5 min) and then re-suspending them in filtered seawater (FSW, 0.22 µm). Bleached, juvenile sea anemones (from a clonal *Exaiptasia pallida* line; ∼2–3 mm in height) were prepared via repeated cold shock treatment until they were completely bleached (Muscatine, Grossman & Doino, 1991). Then, the bleached anemones were maintained in the dark. In order to confirm there was no contamination of *Symbiodinium* cells within the bleached anemone before the infection trial, the anemones were cultured under a photoperiod of 12 h light (∼40 µmol m^−2^ s^−1^): 12 h dark (12L/12D) for one week. This period allowed *Symbiodinium* cells, if any, to replicate within the anemone. The presence of multiple *Symbiodinium* cells would more easily be observed. After the incubation period, the anemones were checked by the fluorescent stereomicroscope (AxioCam SteREO Discovery.V8; Zeiss, Germany) to ensure the absence of *Symbiodinium* cells. The bleached anemones were incubated with the clade C *Symbiodinium* (3 × 10^5^ cells/ml) in a sterile 6-well cell culture plate (10∼15 anemones per well with 1.5 mL of infection solution). After 1 h of co-culturing, most of the anemones were infected with the clade C *Symbiodinium*. The infected sea anemones were washed twice with FSW to remove any *Symbiodinium* cells that were not taken up before being moved to a new tank.

### Culturing of infected anemones and their offspring

Juvenile anemones infected with the clade C *Symbiodinium* were cultured in a tank (GEX GENOAH100, Japan; 10.8 × 10.8 × 12.9 cm; volume: 800 ml, with a transparent cover) under a photoperiod of 12 h light (∼34 µmol m^−2^ s^−1^): 12 h dark (12L/12D) at a temperature of 25 °C ± 1 °C and fed brine shrimp larvae once a week. The FSW in the tanks was changed twice a week. The clade C-infected anemones, which make up the first generation (G1), were maintained in the laboratory for more than one year, during which they matured and underwent asexual reproduction (pedal laceration). The production of lacerates (offspring) from the clade C-infected anemones was checked daily. The newly produced lacerates (the second generation, G2) were moved to a new tank for further development. When the G2 anemones grew up and began to produce lacerates, the lacerates (the third generation, G3) were moved to a new tank or a 6-well plate for further development. Lacerate development of the same specimen was observed in a 6-well plate and recorded by a fluorescent stereomicroscope until they became juveniles (∼9 days). The juvenile anemones were maintained in the same conditions as described above.

### Restriction fragment length polymorphism (RFLP) analysis

For RFLP analysis, three individual clade-C *Symbiodinium* infected anemones or specimens, as well as their offspring, were collected. Several tentacles from both first generation (G1), the clade C *Symbiodinium*-infected anemones and second generation (G2, the offspring of G1) anemones were collected. Since the *Symbiodinium* number in a single tiny anemone of the third generation (G3) was not enough for efficient DNA extraction, 5–10 juvenile anemones were collected, pooled, and processed as a single specimen for analysis. To demonstrate that the anemone which originally harbors clade B *Symbiodinium* can now harbor clade C *Symbiodinium*, DNA from one representative anemone, which is from the same clonal line as the clade C-infected anemone and cultured under the same conditions but not subject to cold shock bleaching, was also analyzed. Genomic DNA of all specimens was extracted using the Plant Genomic DNA Extraction Miniprep System (Cat. GPG1001; Viogene, Taiwan) according to the manufacturer’s recommendations. Genomic fragments from small-subunit ribosomal RNA genes (18S rDNA) were amplified from nucleic acid samples using the *Symbiodinium*-specific PCR primers ss5z and ss3z as previously described (Rowan & Powers, 1991a). Briefly, the DNA amplification was performed in a DNA Thermal Cycler (GeneAmp PCR System 2700; Applied Biosystems) with the following thermal cycles: 1 cycle of 94 °C for 4 min, 55 °C for 30 s, 72 °C for 2 min; and then 29 cycles of 94 °C for 45 s, 55 °C for 30 s, 72 °C for 2 min; plus a final 5 min extension at 72 °C after the 29 cycles. PCR products were purified by High Pure PCR Product Purification Kit (Roche, Germany) and then digested with either the restriction enzyme *Taq* I or *Sau*3A I (New England Biolabs, USA) according to the manufacturer’s recommendations. The digested DNA fragments of each sample were separated by electrophoresis (110 V, ∼20 min) on 1.5% 0.5x TAE buffer (Amresco, USA) agarose gels, to generate the RFLP pattern. Comparing the obtained RFLP patterns with known patterns of *Symbiodinium* B1 and C1 (Rowan & Powers, 1991a; Rowan & Powers, 1991b; Rowan & Knowlton, 1995) enabled the confirmation of the genetic identities of the dinoflagellates.

### Microscopic examination of *Symbiodinium* cells

Three juvenile G3 anemone specimens harboring clade C *Symbiodinium* and three juvenile anemone specimens harboring clade B *Symbiodinium* were anaesthetized with 7% MgCl_2_ in FSW for 5 min. The specimens were then placed on slides and covered with a 22 × 22-mm coverslip for observation with an upright microscope (Axioskop 2 Plus, Zeiss, Germany), and three differential interference contrast (DIC) images of each specimen’s tentacles were obtained. Using the open source, Java-based software package ImageJ (National Institutes of Health, Bethesda, MD, USA), the diameters of all *Symbiodinium* cells in each image were measured, and the average *Symbiodinium* cell size was calculated. A student’s *t*-test was used to identify differences between the two groups, which resulted in a *p* value < 0.01 indicating significance. All results have been expressed as mean ± SD. For reference, free-living cultured clade B and C *Symbiodinium* were centrifuged to concentrate the cells. Aliquots of each *Symbiodinium* solution was dropped on slides for imaging and cell size measurement as above.

## Results and Discussion

**Figure 1:**
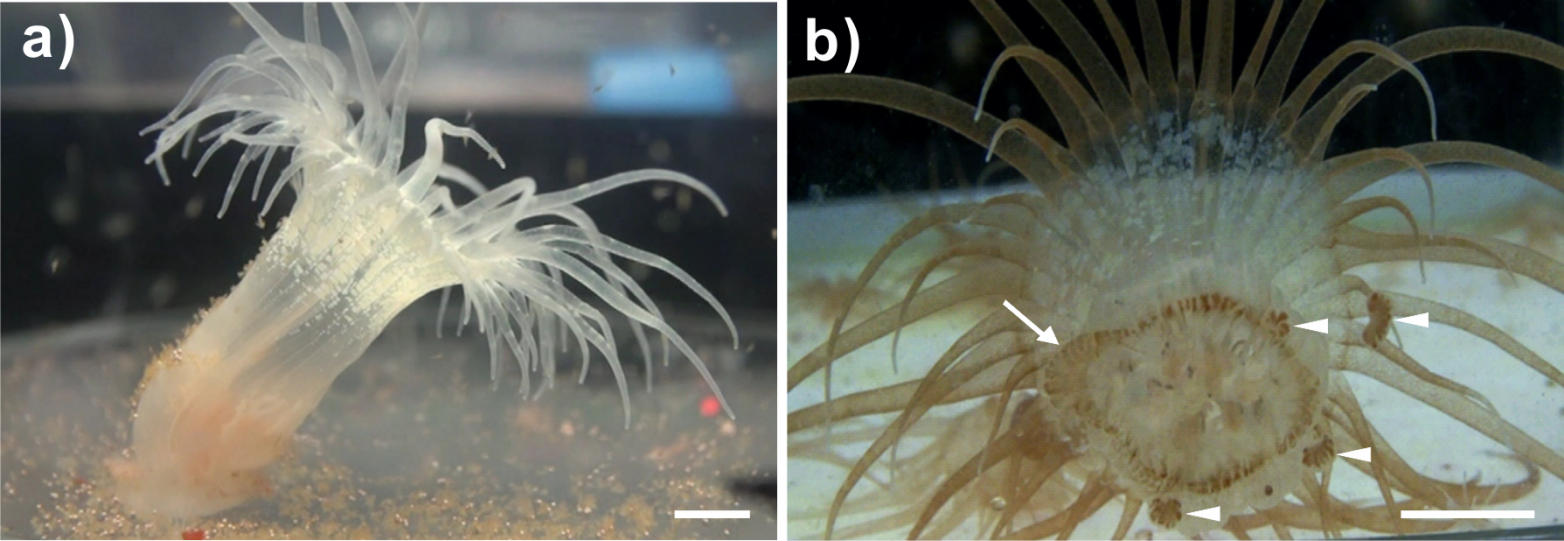
A representative bleached and a representative clade C *Symbiodinium*-infected anemone and its lacerates. (A) Representative image of a bleached anemone that had lost its brownish coloration following the expulsion of *Symbiodinium* cells during cold shock-induced bleaching. (B) Aboral view of a representative clade C *Symbiodinium*-infected anemone showing brownish *Symbiodinium* cells distributed throughout the body, with a notable degree of dinoflagellate aggregation in the margins of the pedal disk (arrow), as well as within the newly budded lacerates (triangles). Scale bars: 0.5 cm.

To determine whether the clade C *Symbiodinium* (CCMP2466; ITS2 type C1) could be taken up by *Exaiptasia* anemones and then be transmitted to offspring after proliferation through the adult tissues, anemones infected with the clade C *Symbiodinium* were cultured for more than one year. During this period, juvenile anemones infected with *Symbiodinium* C1 grew from 2–3 mm in height to 2–3 cm in height, and *Symbiodinium* cells proliferated throughout the bodies of the specimens (Fig. 1B). Asexual reproduction (pedal laceration) was also observed and recorded (Fig. 1B). An aboral view of a representative anemone clearly illustrates the process of asexual reproduction, pedal laceration, in which the newly budded lacerates surround the pedal disk of the anemone (Fig. 1B). Furthermore, *Symbiodinium* cells were found to aggregate within the lacerates (Fig. 1B).

**Figure 2:**
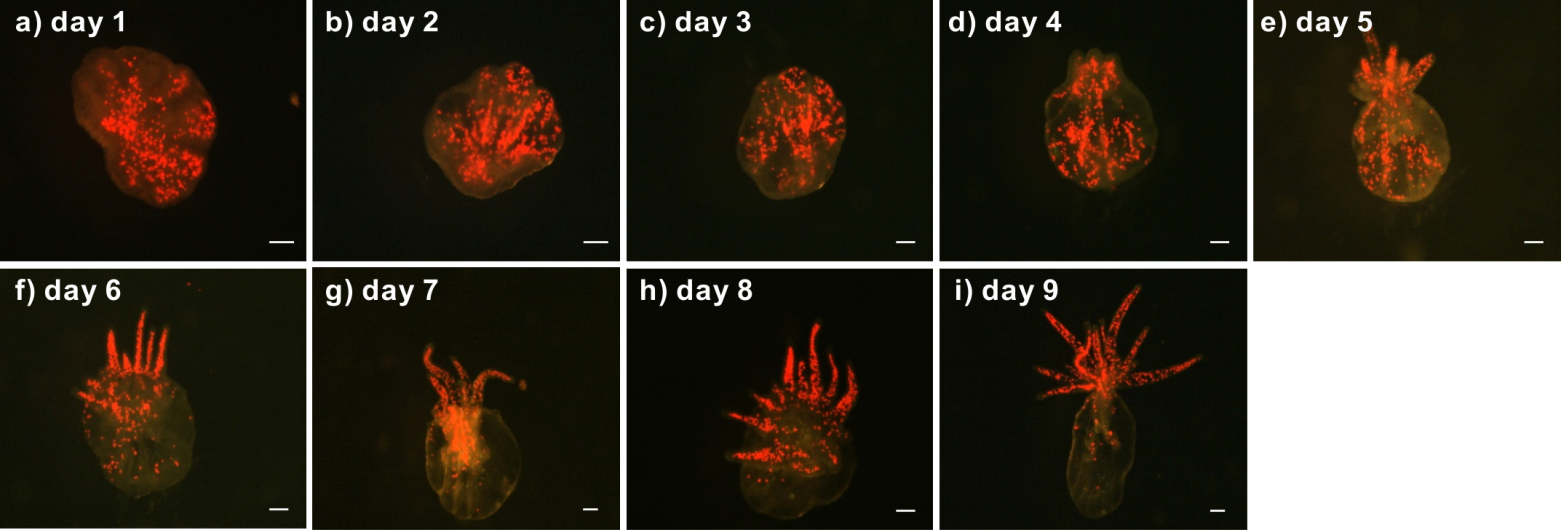
Development of a representative lacerate collected from a clade C *Symbiodinium*-infected anemone. First, the lacerate was transferred to a new dish immediately following laceration from the pedal disk of a clade C *Symbiodinium*-infected anemone. The development of the lacerate and spread of *Symbiodinium* was recorded daily using a fluorescent stereomicroscope (A–I). The red spots in the images indicate chlorophyll autofluorescence of the *Symbiodinium* cells. Scale bars: 100 µm.

The lacerates of *Symbiodinium* C1-infected anemones developed into juvenile anemones within 9 days (Fig. 2). On day 1, red autofluorescence of the *Symbiodinium* cells was already readily observed with a fluorescence stereomicroscope (Fig. 2A). On days 3 and 4, tentacle tissue began forming and extended out from the top of the lacerates, as shown in Figs. 2C and 2D. Between days 5 and 9, the tentacle tissue extended significantly to form the shape of a juvenile anemone (Figs. 2E–2I). During lacerate development, *Symbiodinium* cells were re-distributed to the tentacles, where they are predominantly localized in healthy, adult anemones. Such localization suggests that these clade C *Symbiodinium* cells had successfully established a symbiotic relationship with the juvenile anemones.

**Figure 3:**
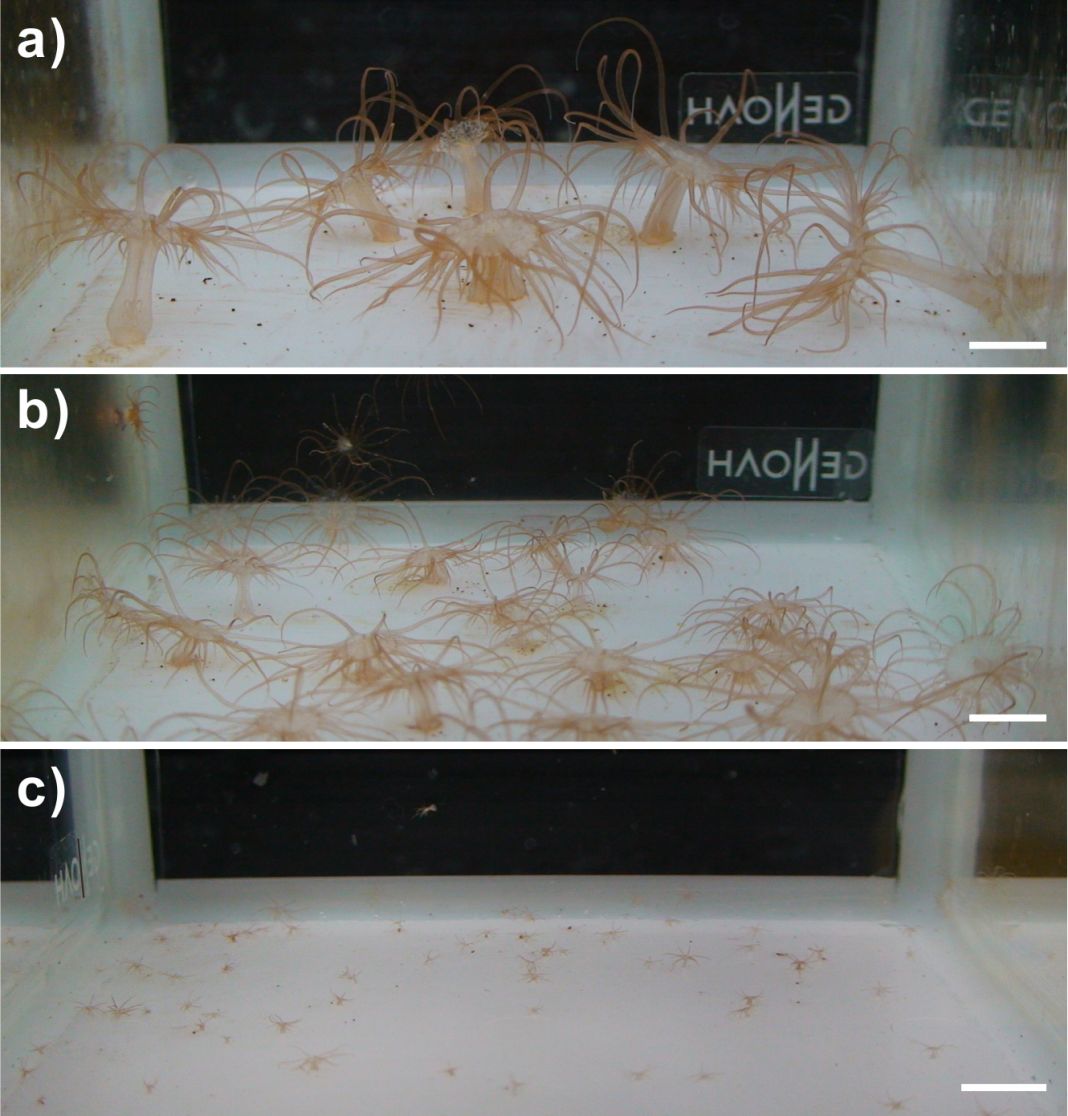
Images of the clade C *Symbiodinium*-infected anemones and their offspring. (A) Generation 1 (G1) anemones maintained in the laboratory for more than one year; (B) Generation 2 (G2) anemones cultured for more than three months; (C) Generation 3 (G3) of anemone cultured for 15 days following laceration. Scale bar: 1 cm.

**Figure 4:**
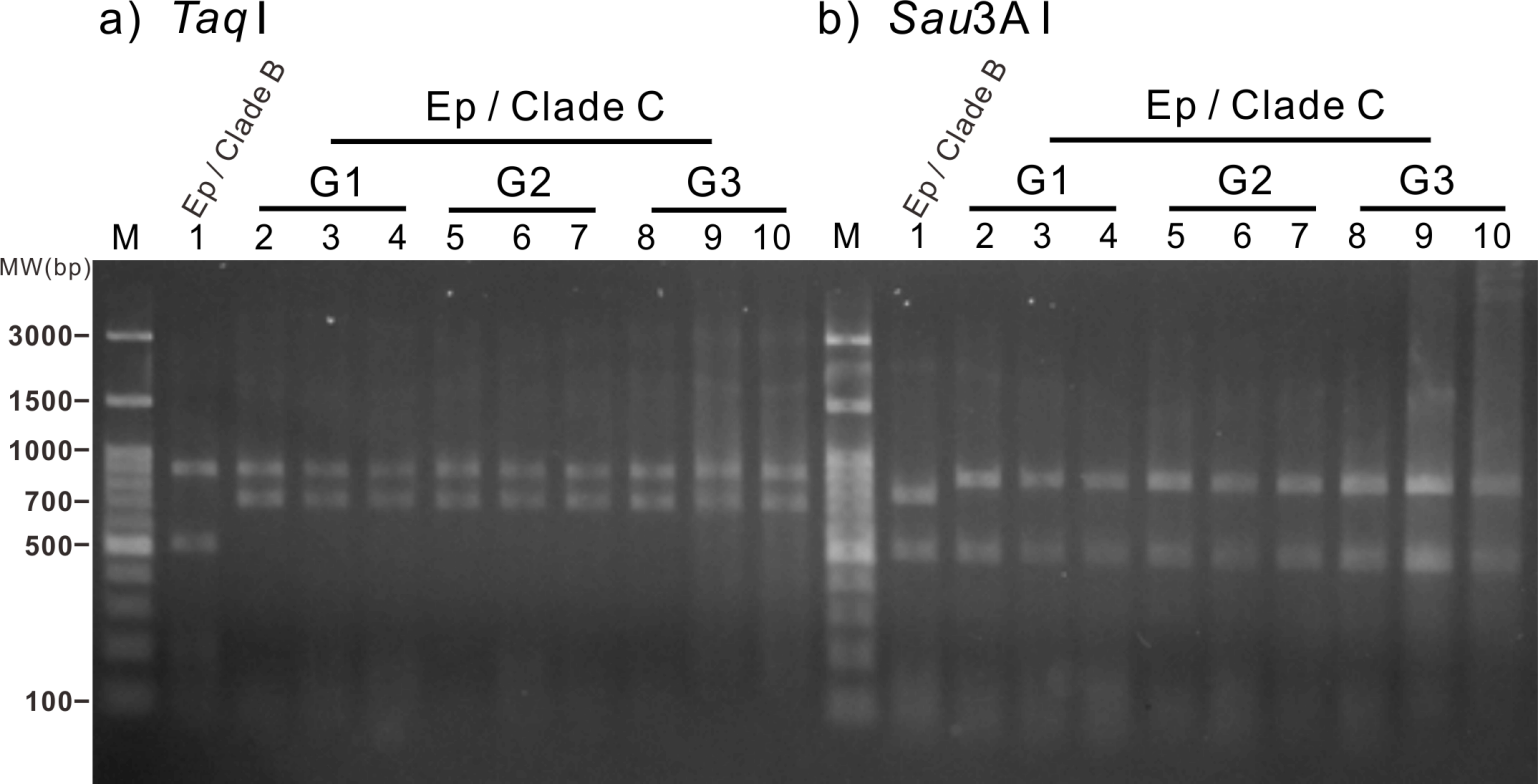
Restriction fragment length polymorphism (RFLP) analysis of three generations of the clade C *Symbiodinium*-infected anemones. Amplified genomic fragments of small subunit ribosomal RNA genes (18S rDNA) from *Symbiodinium* were digested using the restriction enzymes *Taq*I and *Sau*3A I (A and B, respectively). Lane 1 (control): Normal anemone harboring clade B *Symbiodinium*. Lanes 2–4: Generation 1 (G1) anemones. Lanes 5–7: Generation 2 (G2) anemones. Lanes 8–10: Generation 3 (G3) anemones. Ep, *Exaiptasia pallida*; MW, molecular weight.

To confirm the sustainability and genetic identity of the clade C *Symbiodinium* within the infected anemones and their offspring, specimens of three generations of anemones (Fig. 3) were subjected to RFLP analysis of partially digested *Symbiodinium* 18S rDNA. Our results reveal that all three generations of the clade C *Symbiodinium*-infected anemones had the same RFLP pattern (Fig. 4). The major fragments were ∼900 and ∼750 bp when digested with *Taq*I and ∼900 and ∼500 bp when digested with *Sau*3AI (Fig. 4 lanes 2–10); such patterns are diagnostic of *Symbiodinium* C1 (Rowan & Powers, 1991a; Rowan & Powers, 1991b; Rowan & Knowlton, 1995). Furthermore, RFLP patterns of the clade C *Symbiodinium*-infected anemones were well-differentiated from the representative clade B *Symbiodinium*-infected anemone (Fig. 4, Lane 1), which came from the same clonal line as the clade C-infected anemones but was not subject to bleaching and re-infection. The RFLP patterns were ∼900 and ∼500 bp upon digestion with *Taq*I and ∼800 and 500 bp upon digestion with *Sau*3AI (Fig. 4, lane 1). These results clearly demonstrate that the anemones which originally harbored clade B *Symbiodinium* now harbor clade C and transmitted the symbiont for many generations.

**Figure 5:**
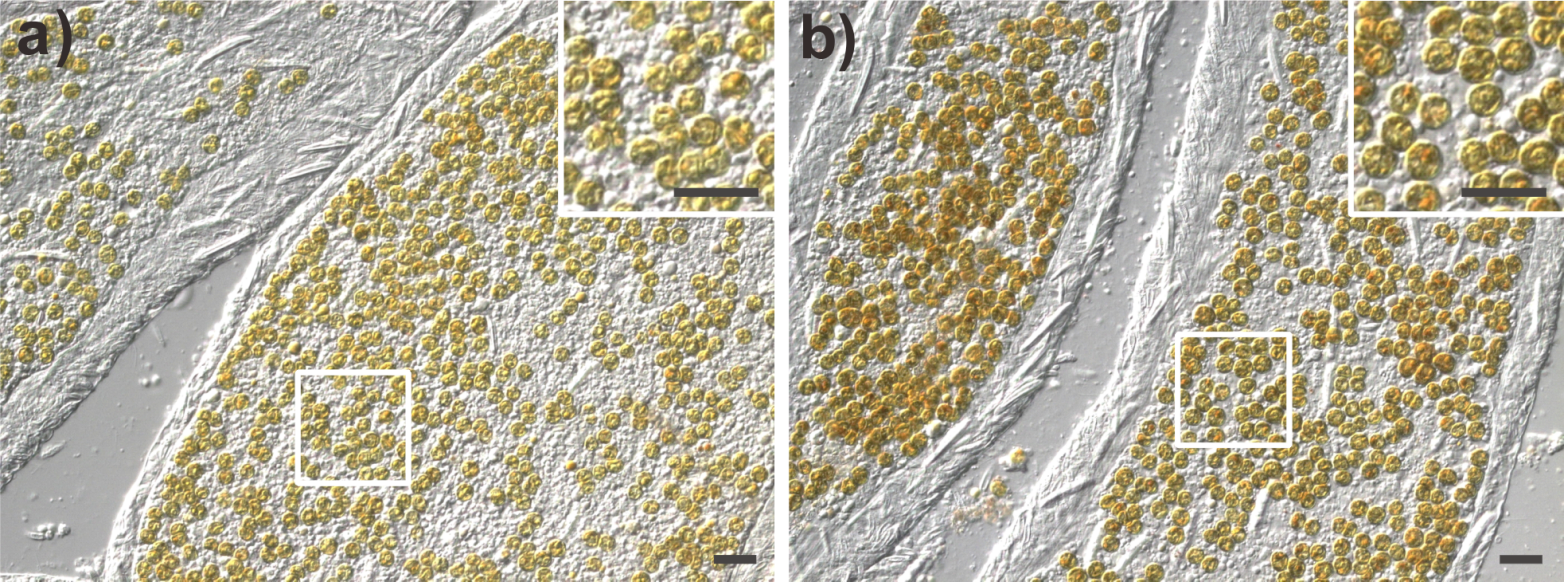
The morphology of clade B and clade C *Symbiodinium* cells within their host anemone tissue. (A) Tentacular tissues harboring clade B *Symbiodinium*; (B) Tentacular tissues harboring clade C *Symbiodinium*. Scale bar: 20 µm.

In addition to the RFLP analysis, the bright field images (Fig. 5) and diameter measurement of *Symbiodinium* further support the presence of clade C *Symbiodinium* cells in the anemones in contrast with the presence of clade B *Symbiodinium*. As shown in Fig. 5, the clade C *Symbiodinium* cells appear more darkly brownish than clade B cells. Moreover, the clade C *Symbiodinium* cells averaged 8.30 ± 0.09 µm (*n* = 855) in diameter *in hospite*, significantly larger (*p* < 0.01) than *in hospite* clade B cells (7.72 ± 0.13 µm, *n* = 992). Since the cell size of the clade C *Symbiodinium* (6.70 ± 0.84 µm, *n* = 1,000) is significantly larger (*p* < 0.01) than clade B *Symbiodinium* (6.00 ± 0.68 µm, *n* = 1,000) in free-living cultures, the larger *Symbiodinium* cells in the anemones demonstrate it harbors the clade C *Symbiodinium* instead of the clade B. It also demonstrates that like the clade B *Symbiodinium* (Pasaribu et al., 2015), the cell size of the clade C *Symbiodinium* increase in endosymbiotic condition. This experimental evidence reveals that the exogenously supplied clade C *Symbiodinium* can be transmitted to multiple generations of progeny via pedal laceration under laboratory conditions. We thus conclude that the *Exaiptasia* anemone species used in this study can establish a symbiotic relationship with the clade C *Symbiodinium* (CCMP2466; ITS2 type C1). In addition, our study and previous work that has infected *Exaiptasia* with heterologous *Symbiodinium* types originally from other host species (Schoenberg & Trench, 1980; Hawkins et al., 2016) also demonstrate that *Exaiptasia pallida* could potentially establish symbiotic relationships with other clades of *Symbiodinium*.

Recently, the scientific name of several anemones used widely as a model system for the study of cnidarian-dinoflagellate endosymbiosis has been revised to *Exaiptasia pallida* (Grajales & Rodriguez, 2014; Grajales & Rodriguez, 2016; Grajales, Rodriguez & Thornhill, 2016). This change was based on the updated morphological and genetic population study of the newly collected specimen from around the world. According to these global comparative investigations, the anemones used in our study, which originate from the same wild population as the studies cited above (N22 03 00.08 E120 41 42.88), belong to a widespread lineage that hosts *Symbiodinium minutum* (ITS2 type B1). These updated studies also found that, in addition to the previously known Florida lineage of *Exaiptasia pallida* which hosts *Symbiodinium* A4, B1, and a mixed population of B1 and C1 (Thornhill et al., 2013), *Exaiptasia* hosts a mixed population of *Symbiodinium* B1 and C1 in Bermuda and *Symbiodinium* type A4 in Mexico and the Bahamas (Grajales & Rodriguez, 2016; Grajales, Rodriguez & Thornhill, 2016). Therefore, although the present study is not the first to document *Exaiptasia* spp. hosting clade C *Symbiodinium*, it does show for the first time that the *Exaiptasia pallida* anemones predominantly known to host the clade B *Symbiodinium* (LaJeunesse, Parkinson & Reimer, 2012; Thornhill et al., 2013; Kinzie et al., 2001) can be bleached and re-infected with exogenously supplied dinoflagellate algae of the clade C *Symbiodinium*.

The clade C *Symbiodinium* (CCMP2466) used in this study was originally isolated from a corallimorph (*Discosoma sanctithomae)*, not a reef-building coral, but the genetic data shows that it belongs to a line (ITS2 type C1; Krueger et al., 2015) of diverse clade C *Symbiodinium* that are mainly harbored by reef-building corals (Van Oppen et al., 2001; LaJeunesse et al., 2003; LaJeunesse et al., 2004b; Chen et al., 2005; LaJeunesse et al., 2010) and are sensitive to thermal stress (Baker et al., 2004; Litmann, Bourne & Willis, 2010). Since CCMP2466 could infect aposymbiotic larvae of a reef-building coral, *Acropora tenuis*, and then establish a monoclonal *Symbiodinium* C1-infected coral association (Yuyama & Higuchi, 2014), CCMP2466 has been successfully applied to the study of thermal tolerance of corals when it harbors the thermal sensitive clade C (CCMP2466) or thermal tolerant clade D *Symbiodinium* (Yuyama et al., 2016). These updated reports and this present study imply that the *Exaiptasia-Symbiodinium* C1 association is an interesting and useful system for studying the functional diversity between cnidarian hosts and their symbionts.

In conclusion, the present study opens up a window for future studies to determine whether the molecular pathways and the type of symbiosis underlying the establishment of the *Exaiptasia*–*Symbiodinium* B1 association are similar to the *Exaiptasia*–*Symbiodinium* C1 endosymbiotic association. If this relationship is later shown to be mutualistic, as is *Exaiptasia-*clade B *Symbiodinium* association, then this could serve as a potentially valuable model for the study of cnidarian-*Symbiodinium* endosymbiosis.

##  Supplemental Information

10.7717/peerj.2358/supp-1 Figure S1 Restriction fragment length polymorphism (RFLP) analysis of free-living cultured clade C and clade B *Symbiodinium* Amplified genomic fragments of small subunit ribosomal RNA genes (18S rDNA) from *Symbiodinium* were digested using the restriction enzymes *Taq*I. Lane 1: The free-living cultured clade C *Symbiodinium* (CCMP 2466). Lane 2: Free-living cultured clade B *Symbiodinium* (originally from *Exaiptasia pallida*). M: marker. MW: molecular weight.

## Additional Information and Declarations

### Data Availability

The following information was supplied regarding data availability: All of the raw data are presented in the manuscript. A supplementary figure has been included as Fig. S1.
