# Supplementary material for: Transmission of a heterologous clade C Symbiodinium in a model anemone infection system via asexual reproduction
Source: PeerJ. 2016 Aug 24;4:e2358. doi: 10.7717/peerj.2358 (PMC5012276; doi:10.7717/peerj.2358)
Supplement: Figure S1 — Amplified genomic fragments of small subunit ribosomal RNA genes (18S rDNA) from Symbiodinium were digested using the restriction enzymes TaqI. Lane 1: The free-living cultured clade C Symbiodinium (CCMP 2466). Lane 2: Free-living cultured clade B Symbiodinium (originally from Exaiptasia pallida). M: marker. MW: molecular weight. [file peerj-04-2358-s001.pdf]

Supplementary Data

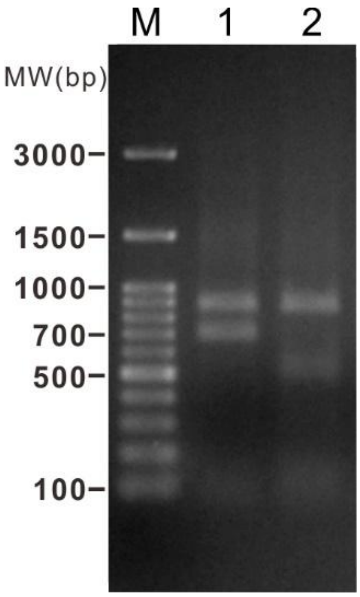

Fig S1. Restriction fragment length polymorphism (RFLP) analysis of free-living cultured clade C and clade B *Symbiodinium*. Amplified genomic fragments of small subunit ribosomal RNA genes (18S rDNA) from *Symbiodinium* were digested using the restriction enzymes *Taq*I. Lane 1: The free-living cultured clade C *Symbiodinium* (CCMP 2466). Lane 2: Free-living cultured clade B *Symbiodinium* (originally from *Exaiptasia pallida*). M: marker. MW: molecular weight.
